# Nimodipine Reduces Dysfunction and Demyelination in Models of Multiple Sclerosis

**DOI:** 10.1002/ana.25749

**Published:** 2020-05-06

**Authors:** Roshni A. Desai, Andrew L. Davies, Natalie Del Rossi, Mohamed Tachrount, Alex Dyson, Britta Gustavson, Pardis Kaynezhad, Lewis Mackenzie, Marieke A. van der Putten, Daniel McElroy, Dimitra Schiza, Christopher Linington, Mervyn Singer, Andrew R. Harvey, Ilias Tachtsidis, Xavier Golay, Kenneth J. Smith

**Affiliations:** ^1^ Department of Neuroinflammation UCL Queen Square Institute of Neurology, University College London London UK; ^2^ Department of Brain Repair and Rehabilitation UCL Queen Square Institute of Neurology, University College London London UK; ^3^ Bloomsbury Institute for Intensive Care Medicine, Division of Medicine University College London London UK; ^4^ Biomedical Optics Research Laboratory, Department of Medical Physics and Biomedical Engineering University College London London UK; ^5^ School of Physics & Astronomy, University of Glasgow Glasgow UK; ^6^ Glasgow Biomedical Research Centre Room B3‐19, 120 University Place, University of Glasgow Glasgow UK; ^7^ Nuffield Department of Clinical Neurosciences John Radcliffe Hospital Oxford UK; ^8^ Department of Chemistry Durham University Durham UK; ^9^ Northern Centre for Cancer Care, Freeman Hospital Newcastle upon Tyne UK

## Abstract

**Objective:**

Treatment of relapses in multiple sclerosis (MS) has not advanced beyond steroid use, which reduces acute loss of function, but has little effect on residual disability. Acute loss of function in an MS model (experimental autoimmune encephalomyelitis [EAE]) is partly due to central nervous system (CNS) hypoxia, and function can promptly improve upon breathing oxygen. Here, we investigate the cause of the hypoxia and whether it is due to a deficit in oxygen supply arising from impaired vascular perfusion. We also explore whether the CNS‐selective vasodilating agent, nimodipine, may provide a therapy to restore function, and protect from demyelination in 2 MS models.

**Methods:**

A variety of methods have been used to measure basic cardiovascular physiology, spinal oxygenation, mitochondrial function, and tissue perfusion in EAE.

**Results:**

We report that the tissue hypoxia in EAE is associated with a profound hypoperfusion of the inflamed spinal cord. Treatment with nimodipine restores spinal oxygenation and can rapidly improve function. Nimodipine therapy also reduces demyelination in both EAE and a model of the early MS lesion.

**Interpretation:**

Loss of function in EAE, and demyelination in EAE, and the model of the early MS lesion, seem to be due, at least in part, to tissue hypoxia due to local spinal hypoperfusion. Therapy to improve blood flow not only protects neurological function but also reduces demyelination. We conclude that nimodipine could be repurposed to offer substantial clinical benefit in MS. ANN NEUROL 2020 **ANN NEUROL 2020;88:123–136**

Modern therapies for multiple sclerosis (MS) have significantly reduced the frequency of relapses in treated patients.^1^ However, when relapses occur there is little evidence that they are any less severe than before therapy, and their treatment has barely advanced over the 6 decades since steroids were first used. Steroids seem to provide little or no long‐term benefit in treating acute optic neuritis, a disease related to MS, and, although steroid administration can curtail the severity and duration of relapses in MS, any small improvement in long‐term outcome^2^ has to be weighed against the adverse side effects of steroid use. The absence of an effective therapy is a concern because relapses can result in permanent and severe loss of function, and a third of patients treated with steroids fail to make a full recovery.^3^ This therapeutic need is further highlighted by the recent finding that the number of early relapses is associated with an increased risk of developing progressive disease and can, therefore, be used to identify those who may benefit from more active acute therapy.^4^

We have previously reported that loss of neurological function at the onset of experimental autoimmune encephalomyelitis (EAE) is due to spinal hypoxia,^5^, ^6^ in conjunction with loss of mitochondrial function,^7^, ^8^ and these challenges correlate with the expression of neurological deficit. Here, we aim to identify the mechanisms responsible for the hypoxia in a common laboratory model of MS (EAE), and, armed with this knowledge, to develop a rational therapy to protect neurological function in acute relapse.

We additionally hypothesized that overcoming hypoxia could confer protection against the demyelination that characterizes both EAE and MS. Reducing demyelination is important because it avoids the associated axonal conduction block and production of symptoms.^9^ It also avoids leaving axons vulnerable to degeneration and permanent loss of function. One type of demyelination, common in the early forming MS lesion, is of the pattern III subtype^10^ now shown to be due to focal tissue hypoxia,^11^, ^12^ and, in this study, we determine whether therapy designed to prevent hypoxia is effective in reducing demyelination not only in EAE but also the pattern III demyelination in a model of the early MS lesion.

Our multidisciplinary approach has been to examine 2 neuroinflammatory demyelinating models of MS in rats, namely a commonly used model of EAE^5^ and a model of pattern III demyelination mediated by intraspinal injection of lipopolysaccharide (LPS).^12^ We hypothesized that reversing spinal hypoxia in these models using nimodipine, a central nervous system (CNS)‐specific vasodilator, would offer dual benefits of reducing disability and preventing permanent structural damage.

## Methods

### *Animal Models*

Experiments were performed in accordance with the UK Animals (Scientific Procedures) Act of 1986, and the ARRIVE guidelines.

#### *Recombinant Myelin Oligodendrocyte Glycoprotein EAE*

EAE was induced in adult female Dark Agouti rats (DA), as previously described,^5^ and animals “immunized” with incomplete Freund’s adjuvant (IFA)^5^ served as controls in all experiments. Briefly, animals received a subcutaneous injection of either an emulsion consisting of 100 μg recombinant myelin oligodendrocyte glycoprotein (rMOG) in IFA, or an emulsion consisting of equivalent volumes of IFA and saline, at the dorsal aspect of the base of the tail (200 μL) while they are under anesthesia (1.5% isoflurane in room air).

#### *Experimental Pattern III Demyelinating Lesion*

The experimental pattern III demyelinating lesion was induced in adult male Sprague Dawley rats. A quarter laminectomy was performed between the T13 and L1 vertebrae while the rats were under deep isoflurane anesthesia (2% in oxygen) and LPS (0.5 μL of 100 ng/μL in saline; *Salmonella enterica abortus equi*; Sigma‐Aldrich, Dorset, UK) microinjected into the right dorsal column at depths of 0.7 and 0.4 mm, followed by an intramuscular injection of Vetergesic. The wound was closed with sutures and sterile wound clips and the animals were monitored daily for any adverse reactions, as previously described.^12^

### *Basic Cardiovascular Physiology*

Animals with EAE (n = 9) and time‐matched IFA controls (n = 7), were anaesthetized (5% isoflurane in room air for induction and 1.5% for maintenance) at the first peak of disease expression for detailed assessment. A rectal temperature probe connected to a homeothermic heating mat enabled maintenance of normothermia (Harvard Apparatus, Cambridge, UK). Transthoracic echocardiography, to determine myocardial function, was performed using a 14 MHz probe scanning at 0 to 2 cm depth (Vivid 7 Dimension; GE Healthcare, Bedford, UK). Aortic blood flow velocities were determined in the aortic arch using pulsed‐wave Doppler. After the echocardiogram, the left common carotid artery and right internal jugular vein were cannulated using 0.96 mm outside diameter PVC tubing catheter (Biocorp Ltd, Huntingdale, New South Wales, Australia). The arterial line was connected to a pressure transducer (Powerlab; AD Instruments, Chalgrove, UK) for continuous monitoring of mean arterial pressure. The venous line was used for subsequent administration of fluids (0.9% NaCl, 10 mL/kg/h). A tracheostomy was achieved using 2.08 mm external diameter polythene tubing (Portex Ltd, Hythe, Kent, UK) to secure and enable suction of the airway. Whole blood (100 μL) was collected from the carotid arterial line into heparinized capillary tubes (Brand GMBH, Wertheim, Germany) for blood‐gas analysis (ABL 90 FLEX, Radiometer, Crawley, UK), including measurement of the partial pressures of oxygen (PaO_2_) and carbon dioxide (PaCO_2_), pH, hemoglobin (quantity, percentage oxygen bound, and calculated p50), glucose, lactate, and electrolytes (K^+^, Na^+^, Ca^2+^, and Cl^−^). At the end of the experiment, animals were euthanized by terminal anesthesia (5%).

### *Spinal Measurements in vivo in EAE*

At the first peak of disease expression, a laminectomy was performed at the T13/L1 vertebral junction (2% isoflurane in room air), and the spinous process of L1 removed to expose the dorsal aspect of the lumbar enlargement, in EAE and IFA time‐matched control animals, as previously described.^5^ A rectal temperature probe connected to a homeothermic heating mat (Harvard Apparatus, Cambridge, UK) enabled maintenance of normothermia throughout the experiment and global arterial oxygen saturation was monitored continuously by pulse oximetry (MouseOx; STARR Life Sciences, Oakmont, PA) via a hind foot sensor. Anesthesia was maintained (1.5% isoflurane in room air) for the duration of the experiment. Where appropriate, photomicrographs were taken of the exposed spinal surface (Nikon D1000 camera).

#### *Spinal Oxygen Measurements*

An oxygen probe (NX‐BF/O/E; 250 μm tip diameter; OxyLite Pro; Oxford Optronix, Oxon, UK) was inserted into the spinal grey matter of EAE (n = 9) and IFA control (n = 7) animals (measurements were taken from the spinal grey matter as the oxygen concentration in the spinal white matter can be too low to detect accurately with the probe), through a small opening in the dura for measurement of spinal tissue oxygen tension (tPO_2_) under light anesthesia (1.5% isoflurane in room air). The fraction of inspired oxygen (FiO_2_) was then increased from 21% to 100% for an additional 10‐minute period. At the conclusion of the experiment, animals were euthanized by terminal anesthesia (5% isoflurane in room air).

#### *Assessment of Spinal Tissue Pressure/Edema*

A measure of spinal/cerebral spinal fluid (CSF) pressure was obtained using calibrated von Frey hairs to determine the force required to depress the dura, in animals with EAE (n = 20) versus time‐matched IFA controls (n = 6).

#### *Broadband Near‐Infrared Spectroscopy in vivo in EAE Animals*

Needle fiber optic probes with a metal ferrule at the end (500 μm diameter) were used as source and detector fibers and positioned on the exposed dorsal surface of the spinal cord at the first peak of disease expression in rats with EAE (n = 11) and time‐matched IFA controls (n = 9) while under light anesthesia (1.5–2% isoflurane) using a micromanipulator. Care was taken to avoid the dorsal vein in the midline, which could interfere with measurements due to the large absorption. A broadband‐near‐infrared spectroscopy (bNIRS) instrument developed in‐house was used to calculate changes in the concentrations of deoxygenated (HHb), and oxygenated hemoglobin (HbO_2_), together with oxidized cytochrome C oxidase (oxCCO), based on the measured changes in light attenuation through the spinal cord (780–900 nm) using the UCLn algorithm every 10 seconds. The concentrations were expressed in μM.cm as the spinal cord tissue differential pathlength factor was not known.

### *Magnetic Resonance Imaging in EAE Animals*

Longitudinal in vivo magnetic resonance imaging (MRI) measurements were performed on a 9.4 T Agilent scanner (Agilent Technologies, Santa Clara, CA) using a volume coil with an inner diameter of 72 mm (Rapid Biomedical GmbH, Rimpar, Germany) for radio frequency (RF) transmission and a 2‐element array coil for the signal reception (Rapid Biomedical). Based on fast localization imaging, the animals were placed in supine position such that both the heart and the volume of interest were included in the volume coil. The surface coil was placed at the lumbar level and a protocol was developed that overcame the technical challenges inherent to MRI of the spinal cord,^13^ including the synchronization of data acquisition with breathing to reduce motion artifacts. Perfusion maps with an in‐plane spatial resolution of 190 × 190 um^2^ at the lumbar level were obtained using an optimized pre‐saturation‐FAIR‐Q2TIPS ASL preparation combined with a reduced field of view readout sequence. The acquisition parameters were: recovery time = 3.2 seconds, TI_1_/TI_2_ = 1.55/1.65 seconds, TE/TR = 20/5,015 ms, and an acquisition time of 25 minutes. Briefly, after baseline magnetic resonance (MR) measurements, female DA rats were randomly allocated to one of two groups, EAE (n = 7) or controls (n = 6). Longitudinal MR scans were performed at different stages of the disease: first peak (12–17 days postimmunization [dpi]), remission (16–19 dpi), and relapse (19–23 dpi). Animals were scanned while under anesthesia (3% isoflurane in room air for induction and 1.5–2% maintenance). During scanning, body temperature was maintained at 37°C with a heating pad, while heart rate, respiration rate, and arterial oxygen saturation were continuously monitored (Small Animal Instruments, Stony Brook, NY). At the conclusion of the experiment, animals were euthanized by terminal anesthesia (5% isoflurane).

### *Therapy in EAE and the Experimental Pattern III Demyelinating Lesion*

The CNS‐selective vasodilator, nimodipine, was used to determine whether (1) spinal oxygen and blood velocity could be increased in EAE, (2) loss of function could be acutely and chronically decreased in EAE, and (3) the extent of demyelination could be decreased or prevented in EAE and in the experimental pattern III MS lesion.

#### *Drug*

Nimodipine (Sigma Aldrich, Darmstadt, Germany), or vehicle (1:4; ethanol:polyethylene glycol [PEG]) was administered by oral gavage (30 mg/kg), intravenously (1 mg/kg), or subcutaneously using implanted osmotic minipumps (30 mg/kg; Alzet, Cupertino, CA), depending on the experiment.

#### *Nimodipine and Spinal Oxygenation*

At the peak of disease (unilateral or bilateral hindlimb paralysis), the left femoral artery was cannulated (2% isoflurane) to allow continuous arterial blood pressure monitoring (Spike2 Software, Cambridge, UK), prior to performing a laminectomy. A small hole was made in the dura approximately 700 μm lateral to the midline in EAE (n = 17) and time‐matched IFA control animals (n = 10), and an oxygen‐sensitive probe (<50 um tip diameter, OxyMicro, World Precision Instruments, Sarasota, FL) inserted into the spinal grey matter (1.5% isoflurane), as previously described.^5^ Oxygen, rectal and body temperatures, arterial blood pressure, and global arterial oxygen saturation were recorded continuously. Following an initial stabilization period of 5 minutes, a bolus of nimodipine (1 mg/kg, EAE: n = 9; IFA: n = 6) or vehicle (1:1:3, ethanol: PEG400: saline; EAE: n = 8; IFA: n = 4) was injected intravenously into the tail vein. Recordings were continued for up to 1 hour following the intravenous injection, until the spinal oxygen concentration stabilized.

#### *Nimodipine and Blood Velocity*

Animals with EAE were selected for imaging at the first peak of disease and examined in conjunction with time‐matched IFA controls (EAE: n = 17; IFA: n = 9). The spinal cord beneath the L2 vertebra was exposed by laminectomy (2% isoflurane in room air), and the vertebral column stabilized using side clamps. Rectal temperature was maintained at 35.5 ± 2°C using a homeothermic underblanket. The dura was left intact and protected with warmed mineral oil. The cord was illuminated using an X‐Cite lamp and viewed using an FITC filter (Filter Set 10; Zeiss United Kingdom, Cambridge, UK). A full resolution image was obtained with a charge‐coupled device camera set to 1,000 ms exposure time (QImaging, Surrey, Canada). Fluorescent microspheres (1.9 μm; ThermoFisher Scientific, Paisley, UK) were administered via cannulation of the femoral vein and imaged at 10 Hz, binning pixels at 4 × 4. A bolus of nimodipine (1 mg/kg, EAE: n = 10; IFA: n = 5) or vehicle (1:1:3, ethanol:PEG400:saline, EAE: n = 7; IFA: n = 4) was administered intravenously. Spinal blood flow was monitored for 45 minutes following the intravenous injection, with a full resolution image and a 10 Hz recording taken at 5, 15, 30, and 45 minutes postinjection. Velocity at each time point was determined by typically making >40 measurements of streak length (distance travelled by a microsphere in one frame) and 10 measurements of vessel diameter per recording. Images were acquired using MicroManager and analyzed in ImageJ.

#### *Nimodipine and Acute Loss of Function in EAE*

EAE animals with a range of deficits were randomly allocated to receive nimodipine (30 mg/kg: n = 30) or vehicle (1:4, ethanol:PEG400; n = 18) via oral gavage, based on their 10‐point neurological score. The scoring system comprised of points assigned based on the deficit observed, with one point assigned for tail‐tip weakness, tail‐base weakness, tail paralysis, absence of toe spreading reflex, abnormal gait, hind limb paresis (left and right), hind limb paralysis (left and right), and moribund.

Rectal temperature was measured before and after nimodipine administration to explore whether the improvement in neurological function may be due to a Uhthoff effect^9^ due to body cooling.

#### *Continuous Nimodipine and Disease Course in EAE*

At the onset of neurological deficit, animals were randomly allocated to receive either nimodipine (30 mg/kg; n = 13) or vehicle (ethanol: PEG400; n = 13) via subcutaneously implanted osmotic minipumps (Alzet, Cupertino, CA). Animals were evaluated daily for their neurological deficit, in a blinded manner, until termination 12 days post‐onset of neurological deficit. Animals were perfused transcardially with rinse followed by paraformaldehyde (PFA),^5^ and the spinal cord harvested for histological evaluation. The gastrocnemius muscle was also harvested and weighed.

#### *Nimodipine and Experimental Pattern III Demyelination*

Pattern III demyelinating lesions were induced as described above. Following surgery, animals received an intraperitoneal (i.p.) injection of nimodipine (30 mg/kg; n = 20) or vehicle (ethanol:PEG400; n = 19). Animals were allowed to recover and were administered either nimodipine or vehicle via oral gavage at 10, 24, 36, and 48 hours postinjection. Animals were monitored daily for any adverse effects and terminated via transcardial perfusion 14 days postinjection. Spinal cords were harvested and processed for histological evaluation.

### *Histology and Microscopy*

Harvested spinal cords were post‐fixed in 4% PFA for 24 hours, with subsequent cryoprotection in 30% sucrose (Sigma, Welwyn Garden City, UK) in phosphate‐buffered saline (PBS). The relevant regions of the cords (EAE: lumbar enlargement; pattern III lesion: injection site marked with charcoal) were processed for cryosectioning and stained with Luxol fast blue, periodic acid Schiff, and hematoxylin (LFB/PAS/H) to assess demyelination. Every tenth section from 200 sections (∼2,400 μm lesion) of pattern III lesioned tissue was stained and examined to identify the lesion epicenter. Demyelination was then quantified using a section taken at the lesion epicenter. Tissue was viewed using an Axiophot light microscope (Carl Zeiss, Oberkochen, Germany) and photographed with a Nikon D300 camera (Nikon Instruments, Melville, NY).

### *Histological Quantification*

Quantification of white matter demyelination in EAE was achieved by counting the number of pixels above a set threshold and expressed as the percentage area coverage of the spinal white matter. Lesion size in LPS‐injected animals, as determined by myelin loss in LFB‐stained sections was manually circumscribed and expressed as a percentage of dorsal column area at the lesion epicenter. One section per animal was used for the quantification of demyelination. All quantification was carried out in a blinded manner.

### *Statistics*

Data are presented as mean ± standard error or median, quartiles, and range. Group sizes are indicated in the text. Parametric data (following Shapiro–Wilk testing for normality) were analyzed using repeated measures 1‐way or 2‐way analysis of variance (ANOVA) followed by Bonferroni’s post hoc testing, or an unpaired *t*‐test, as indicated. The Bonferroni correction was applied to adjust the critical level for significance where multiple comparisons were conducted in order to control the family‐wise error rate. Nonparametric data were analyzed using the Mann–Whitney *U* test, or Spearman’s correlation, as indicated. All statistical analyses were 2‐tailed and performed using Prism 7.0.1 software (GraphPad Software, la Jolla, CA). Multiplicity adjusted *p* values <0.05 were considered statistically significant.

## Results

### *Spinal Inflammation is Associated with Tissue Hypoxia*

**Figure 1:**
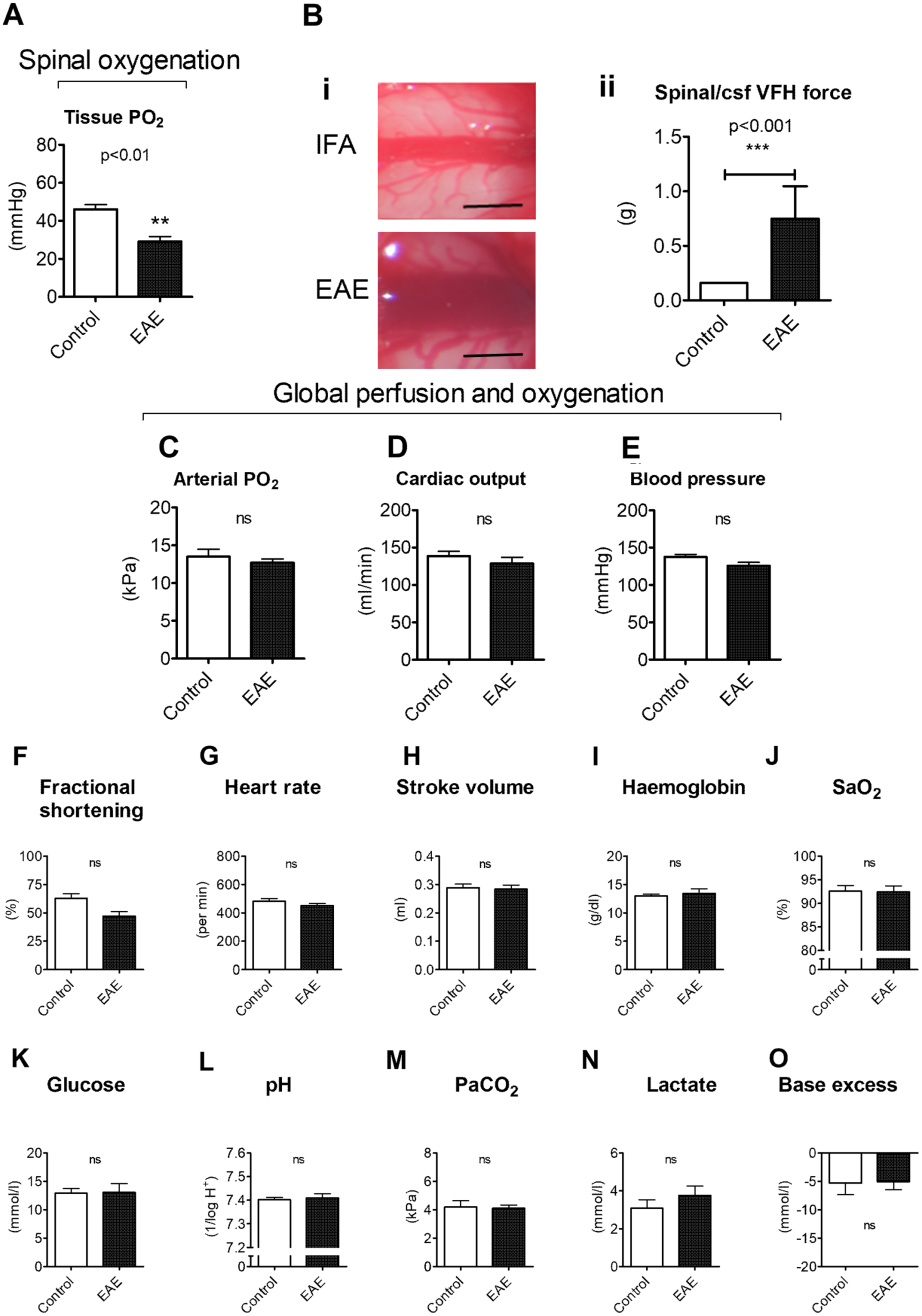
Spinal oxygenation, edema, and global cardiorespiratory function. (A) Shows the spinal cord tissue PO_2_ measured by a fiber‐optic oxygen‐sensitive probe. (B) The i and ii show representative images of the exposed spinal cord and graphical representation of spinal edema in EAE and IFA controls. (C) Shows arterial PO_2_ obtained by blood gas analysis. Echocardiography‐derived cardiac output and carotid arterial mean blood pressure measurements are depicted in (D) and (E), respectively. Echocardiography‐derived fractional shortening, heart rate and stroke volume are shown in panels (F–H). Additional determinants of oxygen delivery, glycemic status, and global indicators of organ perfusion are shown in (I, J, K, L, M, N, and O, respectively). Data presented as mean ± SEM. **p < 0.01, ***p < 0.001, unpaired *t*‐test with Bonferroni correction (A: EAE: n = 8; IFA: n = 7; C–H: EAE: n = 8; IFA: n = 6; I–O: EAE: n = 8; IFA: n = 7), Mann–Whitney *U* test (Bii: EAE: n = 20; IFA: n = 6). CSF = cerebrospinal fluid; EAE = experimental autoimmune encephalomyelitis; IFA = incomplete Freund’s adjuvant; PO_2_ = partial pressure of oxygen; PaCO_2_ = partial pressure of carbon dioxide; SaO_2_ = oxygen saturation; VFH = Von Frey Hairs.

Two independent measurements indicated hypoxia in the spinal cord of animals with EAE. Direct measurement of tissue oxygen tension (tPO_2_) in the spinal cord grey matter using a fiber‐optic oxygen‐sensitive probe (Oxford Optronix, Abingdon, UK) in anesthetized rats showed that those with EAE had significantly lower tissue oxygen tensions (tPO_2_ = 29.01 ± 2.78 mmHg; n = 9) than in controls “immunized” with IFA (45.94 ± 2.47 mmHg; n = 7; unpaired *t*‐test with Bonferroni correction for multiple comparisons: *p* < 0.005; Fig 1A).

### *Spinal/CSF Pressure is Associated with Neurological Deficit*

Photographs of the exposed lumbar spinal cord revealed that the venous blood in animals with EAE appeared more deoxygenated than the venous blood in time‐matched control IFA animals (Fig 1B). The spinal cord in the animals with EAE also appeared to be edematous, and this correlated with the dorsal vein appearing wider, as if it had been flattened beneath the pia. To gain a measure of potential pressure changes in the lumbar cord we used calibrated von Frey hairs to determine the force needed to depress the dura. The results revealed that the force required was significantly larger in animals with EAE (n = 20) than in IFA controls (n = 6) (Mann–Whitney *U* test, U = 3.00, Z = −3.553; *p* < 0.001; Fig 1Bii). The force required to depress the dura was also significantly correlated with the severity of the neurological deficit (Spearman’s correlation, r = 0.710; *p* < 0.001; 95% confidence interval [CI] = 0.436–0.864).

### *Hypoxia is CNS‐Specific, Rather than Systemic*

We next assessed global cardiorespiratory function to examine whether the spinal hypoxia in EAE was due to a systemic reduction in oxygen delivery. Global oxygen delivery is a function of the quantity and saturation of hemoglobin, and cardiac output. Arterial blood gas analysis revealed no differences in the arterial partial pressure of oxygen (arterial PO_2_; Fig 1C), blood hemoglobin levels, or hemoglobin oxygen saturation between animals with or without EAE. Cardiac output showed no difference between treatment groups (Fig 1D), and, at the conclusion of surgery (when the regional assessment of spinal oxygenation took place), blood pressure was also similar in both sets of animals (IFA controls: 137.6 ± 3.33 mmHg, EAE: 125.7 ± 4.68 mmHg; Fig 1E). Although the values for fractional shortening were slightly lower in animals with EAE compared with controls (IFA controls: 62.7 ± 4.20%, EAE: 47.1 ± 4.16%), the reduction was not significant (Bonferroni correction, unpaired t‐test: *t* (12) = 2.57, *p* = 1.00; Fig 1F). Furthermore, echocardiography revealed normal myocardial function, with heart rate and stroke volume showing similar values between animals with or without EAE (Fig 1G,H). No deficits were observed for blood sugar levels, the partial pressure of CO_2_, or any other global indicators of organ hypoperfusion (such as lactate levels or acid/base status; Fig 1F–O). These data argue against a global cardiorespiratory abnormality in animals with EAE during the first peak of disease, and, thus, the severe neurological deficits that occur in conjunction with significant spinal hypoxia are likely to be driven by mechanisms within the spinal cord.

### *Oxygen Administration Overcomes Spinal Hypoxia and Improves Mitochondrial Function*

**Figure 2:**
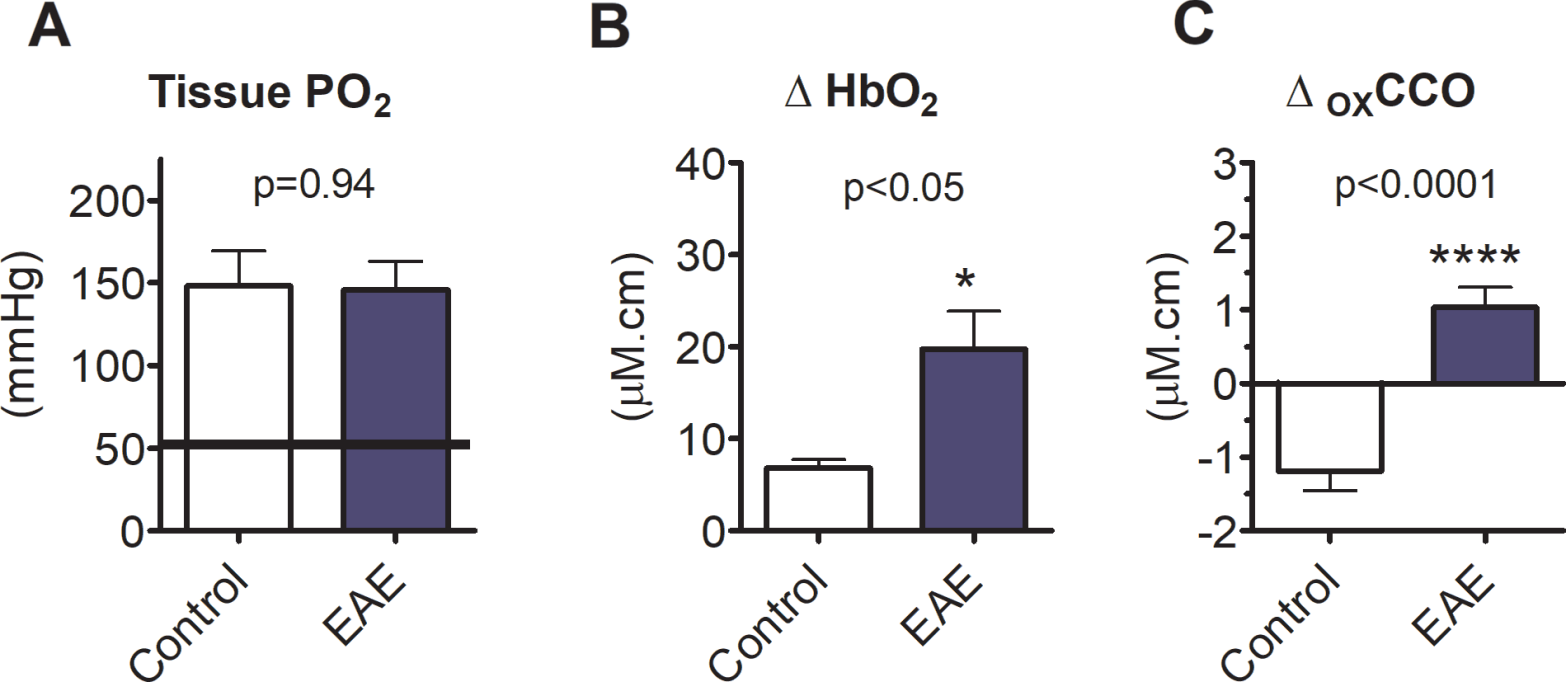
Oxygen administration overcomes spinal hypoxia and improves mitochondrial function. (A) Shows spinal cord tissue PO_2_ measured by fiber‐optic probe following administration of high oxygen concentrations. The grey shaded area shows the normal range obtained in IFA controls breathing room air. (B and C) These panels respectively show changes derived from trans‐spinal broadband near‐infrared spectroscopy in oxyhemoglobin and oxidation of cytochrome C oxidase. Data presented as mean ± SEM. *p < 0.05, ****p < 0.0001; unpaired *t*‐test (A: EAE: n = 8; IFA: n = 7; B and C: EAE: n = 11; IFA: n = 9). EAE = experimental autoimmune encephalomyelitis; HbO_2_ = oxygenated hemoglobin; IFA = incomplete Freund’s adjuvant; oxCCO = oxidation status of cytochrome C oxidase; PO_2_ = partial pressure of oxygen. [Color figure can be viewed at www.annalsofneurology.org]

Given that the inflamed spinal cord is hypoxic, we sought to determine whether inspiratory hyperoxia could overcome spinal hypoxia. Administration of high inspired oxygen concentrations (70% O_2_) elevated tissue oxygen tension beyond the normal range in animals with EAE, as determined by an oxygen‐sensitive probe (Oxford Optronix, Abingdon, UK) inserted into the spinal grey matter (Fig 2A).

We additionally used trans‐spinal bNIRS to examine not only the relative changes in spinal blood hemoglobin oxygenation, but also the oxidation status of mitochondrial cytochrome‐C‐oxidase (a measure of mitochondrial function) upon inspired normobaric hyperoxia (98.5% O_2_). Expectedly, hyperoxia increased the mean spinal oxygenated hemoglobin in animals during the first peak of neurological deficit due to EAE; the magnitude of this increase was significantly higher in EAE (n = 11) versus IFA (n = 9) controls (Fig 2B). Additionally, whereas increasing inspired oxygen did not cause further oxidation of cytochrome‐C‐oxidase in IFA controls, there was a significant increase in the oxidation status of cytochrome‐C‐oxidase in animals with EAE (Fig 2C). This finding indicates an increase in mitochondrial oxygen utilization, and, importantly, this result suggests that whereas spinal mitochondria have a sufficient supply of oxygen in control animals, their function is restricted by an inadequate oxygen supply in EAE. Thus, the loss of neurological function during the first peak of neurological deficit in EAE can be reasonably attributed, at least in part, to tissue hypoxia and an energy crisis arising from mitochondrial deficits. Alleviating this deficit by breathing oxygen improves mitochondrial function, and this is associated with an improvement in neurological function, as reported previously^5^ and confirmed in this study (results not shown).

### *Neurological Deficits and Spinal Hypoxia are Associated with Spinal Hypoperfusion*

**Figure 3:**
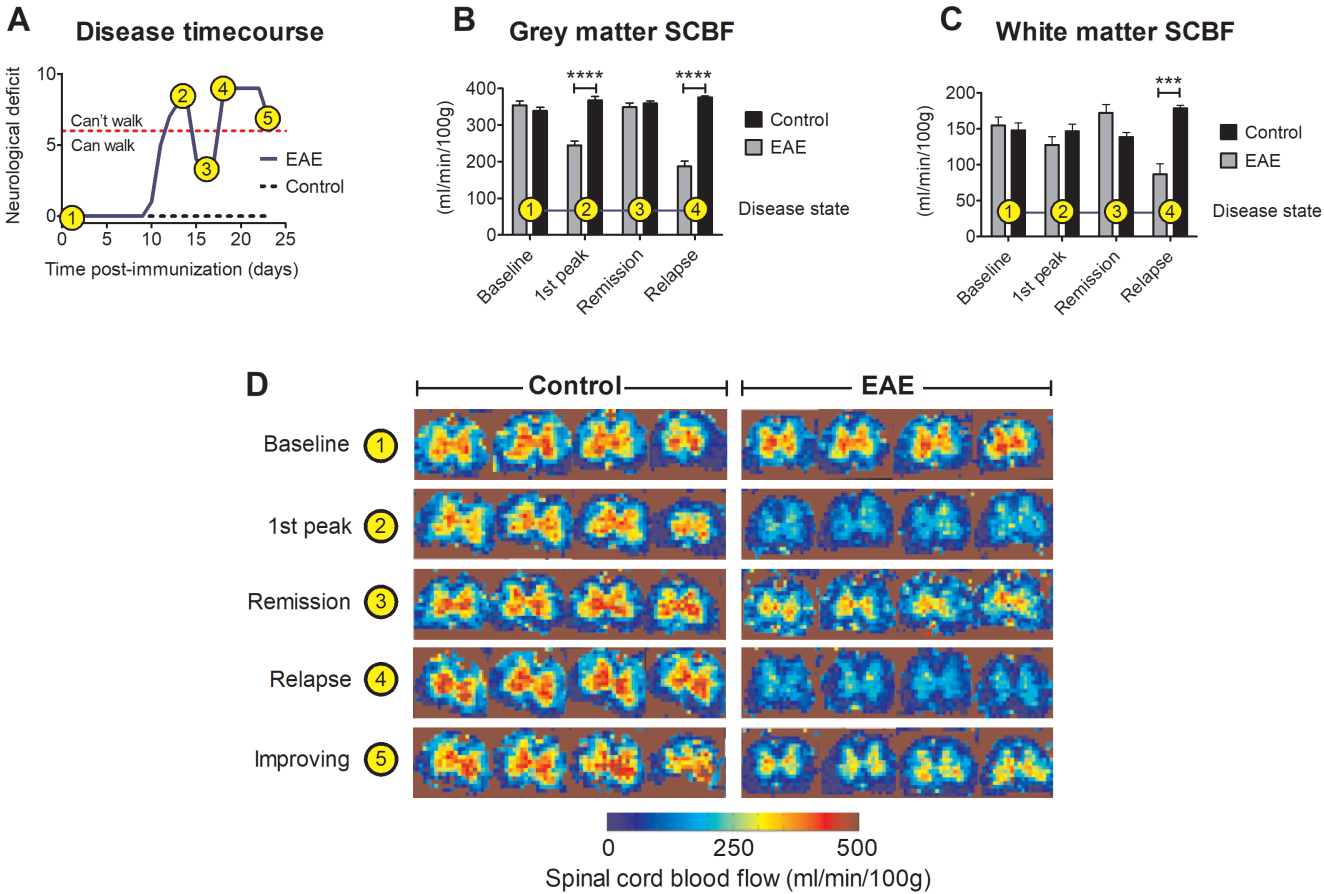
Spinal cord blood flow. The disease time course of an EAE animal is shown in (A) with the following events indicated; 1, baseline (pre‐immunization); 2, first peak of disease; 3, remission; 4, relapse; and 5, post‐relapse improvement of symptoms. The animal is unable to walk using its hind limbs at a neurological deficit >6. SCBF in the grey matter and white matter is shown in (B) and (C), respectively. (D) Shows typical heat maps of SCBF for control and EAE rats at different time points during the disease course. Although the disease course in A is representative of this EAE model up to day 22, an unusual and unexpected improvement was observed on day 23 (indication point 5; improving). This improvement correlates with an increase in spinal blood flow indicated in the MRI heat map in D. Data in B presented as mean ± SEM. ***p < 0.001, ****p < 0.0001; 2‐way repeated measures ANOVA plus Bonferroni’s multiple comparison test (EAE: n = 7; IFA: n = 6). ANOVA = analysis of variance; EAE = experimental autoimmune encephalomyelitis; MRI = magnetic resonance imaging; SCBF = spinal cord blood flow.

We next explored whether the spinal hypoxia and neurological deficits are due to spinal hypoperfusion, and/or driven by increased oxygen demand in the inflamed tissue. We, therefore, made absolute quantitative measurements of spinal cord blood flow (SCBF) in rats with EAE using a novel MRI technique based on arterial spin labeling (ASL). Animals were scanned at different stages of disease, as reflected in the disease time course in Fig 3A; baseline/pre‐immunization (EAE n = 7, IFA control n = 6), peak of first attack (EAE n = 7, IFA control n = 3), remission (EAE n = 6, IFA control n = 3), and relapse (EAE n = 7, IFA control n = 3).

Average pre‐immunization SCBF in grey and white matter was 347 ± 26 mL/100 g/min and 152 ± 23 mL/100 g/min, respectively (Fig 3B,C). Subsequently, SCBF in grey matter was significantly (2‐way ANOVA, *F* (3, 34) = 23.9, *p* < 0.0001; Fig 3B) decreased in animals with EAE during both the first peak of disease (↓ 31%, *p* < 0.001, 95% CI = −29.8 to 61.0) and relapse (↓ 47%, *p* < 0.001, 95% CI = 242–135). Notably, blood flow recovered during remission, and during an unusual improvement in function in one animal on day 23 postimmunization (Fig 3A–D). Within the white matter, the only significant change in SCBF was observed at relapse, where a reduction in flow of 43% (2‐way ANOVA, *F* (3, 34) = 7.60, *p* < 0.001, 95% CI = −145 to 38.3) was observed (Fig 3C. Thus, there was a strong association between reduced spinal cord perfusion and loss of neurological function. We conclude that the neurological deficits at the first peak of neurological deficit in EAE are due, at least in part, to spinal hypoxia arising from hypoperfusion of the inflamed spinal cord.

### *Therapy with Nimodipine Improves Spinal Blood Flow and Tissue Oxygenation*

In view of the finding that reduced spinal cord blood flow and reduced oxygenation accompany spinal hypoxia and loss of neurological function, we next explored whether the L‐type calcium channel blocking agent nimodipine, which selectively dilates CNS blood vessels and increases cerebral blood flow,^14^ is effective in restoring vascular perfusion, spinal oxygenation, and neurological function in animals with EAE.

**Figure 4:**
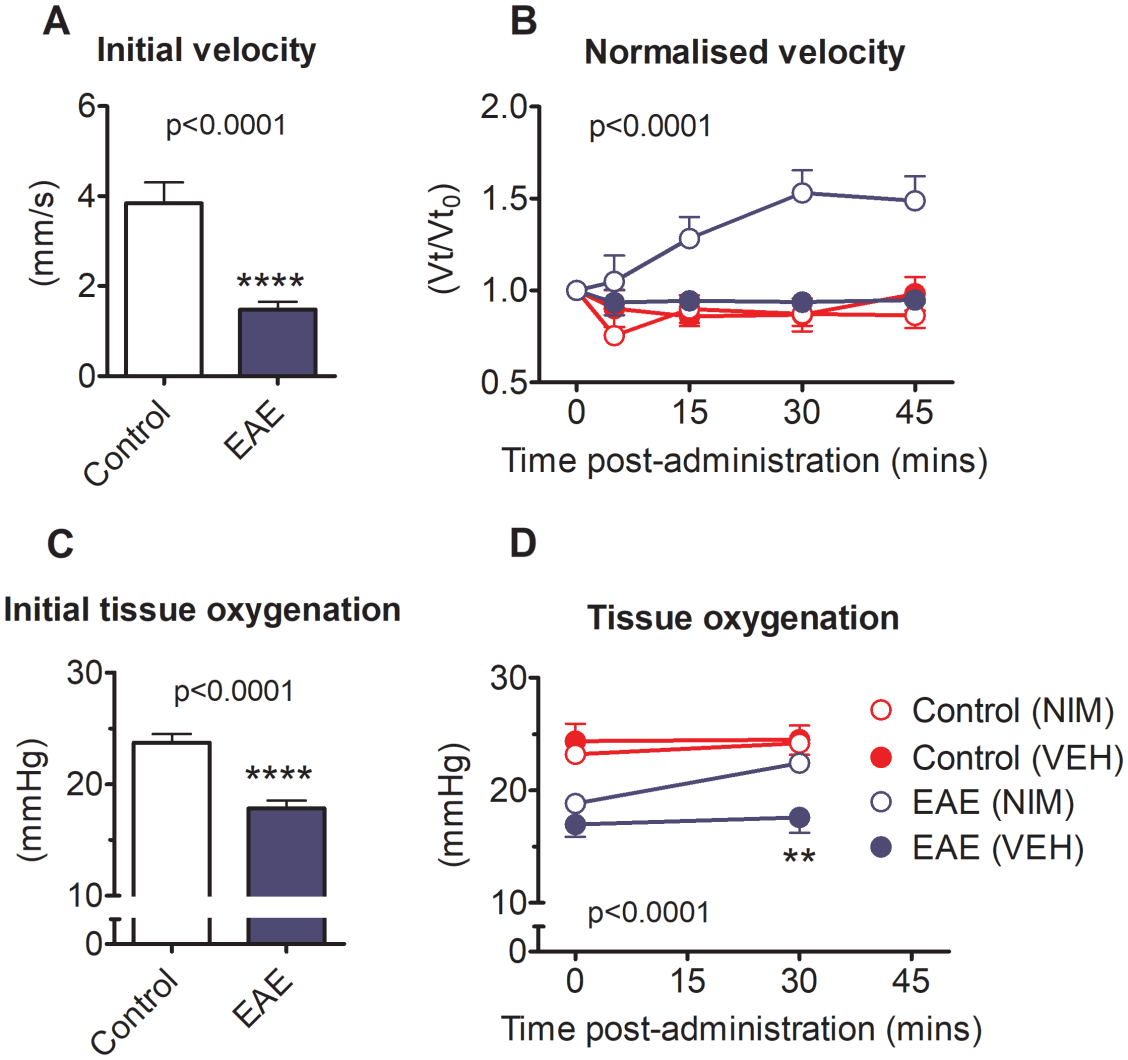
Effect of nimodipine on spinal cord blood flow and oxygenation. (A) Shows initial SCBF velocity in animals with EAE or controls. (B) Shows changes in velocity normalized to each animal’s baseline velocity following treatment with either vehicle or nimodipine. Velocities were measured in the dorsal spinal vein of anesthetized rats and revealed by fluorescent microspheres suspended in the plasma and viewed through the intact dura. (C) Shows spinal tissue oxygenation in control animals compared with animals with EAE. (D) Shows the change in spinal oxygenation in animals with EAE or controls prior to and following the administration of nimodipine or vehicle. Nimodipine improves spinal oxygenation in animals with EAE from hypoxic to normoxic levels. Data presented as mean ± SEM. Stated *p* values reflect the outcome of an unpaired *t*‐test in A and C (****p < 0.0001) and following a 2‐way repeated measures ANOVA in B and D. **p < 0.01; Bonferroni’s multiple comparison test (A, B: EAE + drug n = 10, EAE + vehicle n = 7, IFA + drug n = 5, IFA + vehicle n = 4; C, D: EAE + drug n = 9, EAE + vehicle n = 8, IFA + drug n = 6, IFA + vehicle n = 4). ANOVA = analysis of variance; EAE = experimental autoimmune encephalomyelitis; NIM = nimodipine; SCBF = spinal cord blood flow; VEH = vehicle. [Color figure can be viewed at www.annalsofneurology.org]

The impact of nimodipine on spinal blood flow velocity was measured by imaging fluorescent intravenous microspheres in the dorsal spinal vein of anesthetized rats (Supplementary Movie S1). Imaging was performed in the L2 region in animals with EAE, and in time‐matched controls. Initial blood flow velocity was significantly (*p* < 0.0001) reduced in animals at the first peak of disease expression in EAE compared with IFA controls (Fig 4A,). Following intravenous nimodipine, blood flow velocity was significantly (2‐way ANOVA, *F* (3, 107) = 65.08, *p* < 0.0001, overall ANOVA) enhanced in animals with EAE (n = 10), but had no effect in controls (n = 5; Fig 4B). Vehicle administration had no effect on spinal cord blood flow velocity in either group (n = 7, EAE; n = 4, control; Fig 4B).

In separate studies, animals of equivalent disease severity (hindlimb paresis or paralysis) at the first peak of EAE were anesthetized and an oxygen‐sensitive probe (WPI) inserted into the spinal grey matter at the T13 vertebra. In line with the reduction in blood flow velocity, and our previous findings,^5^ tPO_2_ at the start of the experiment was significantly reduced in EAE animals (n = 17; unpaired *t*‐test, *t* (24) = 5.46, *p* < 0.0001, 95% CI = 3.65–8.10) compared with IFA controls (n = 9; Fig 4C). Intravenous administration of nimodipine, although inducing transient systemic hypotension, was accompanied by a sustained (up to 30 minutes) significant increase in spinal tPO_2_ in animals with EAE (n = 9; paired *t*‐test, *t* (8) = −5.97, *p* < 0.001, 95% CI = −4.79 to −2.12; Fig 4D). Notably, the spinal oxygen concentration in rats with EAE reached control spinal oxygen levels, following nimodipine. No change in spinal tPO_2_ was observed in IFA control animals given nimodipine (n = 5; paired *t*‐test, *t* (4) = −1.16, *p* = 0.311, 95% CI = −3.40 to 1.40) or in either set of animals administered vehicle (IFA control: n = 4; EAE, n = 8; Fig 4D).

### *Acute and Chronic Nimodipine Therapy Restores Function*

**Figure 5:**
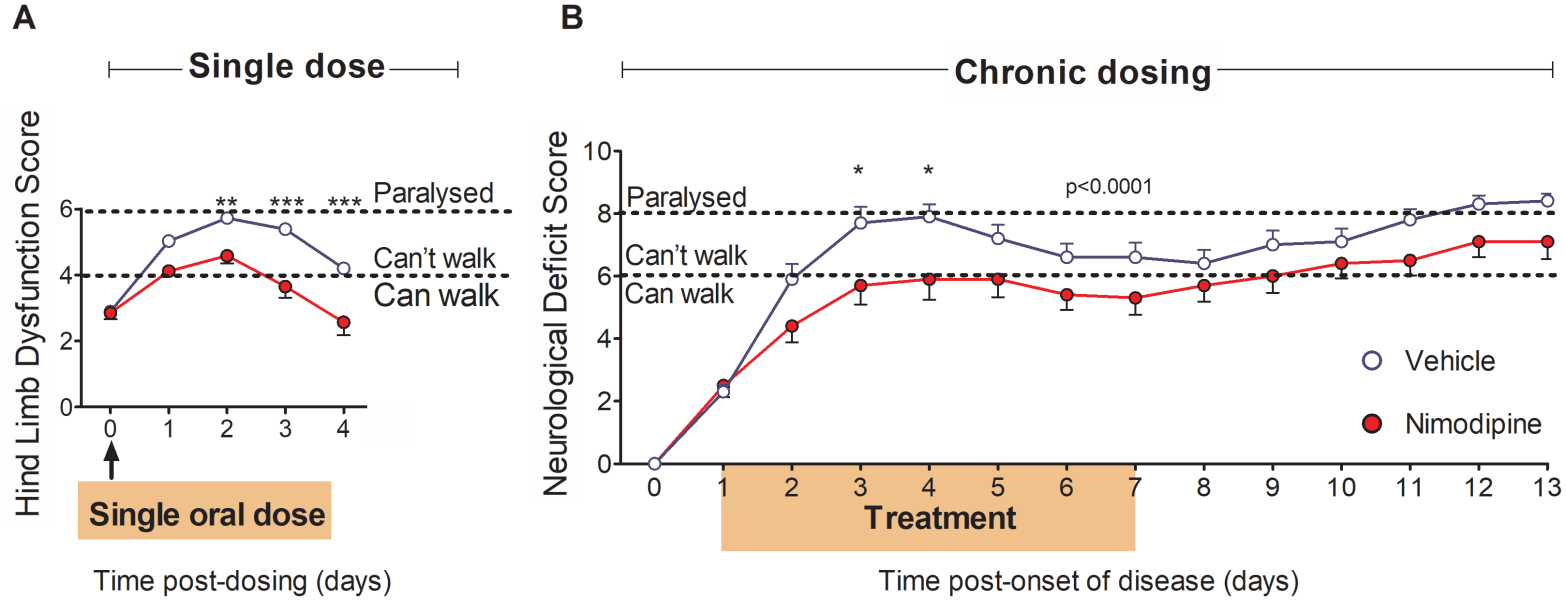
Restoration of function following acute and chronic nimodipine administration. (A) Shows the score for hindlimb dysfunction (one point assigned for gait, toe spreading, hind limb paresis [left and right] and hind limb paralysis [left and right]) in the 4 days following a single administration of vehicle or drug. (B) Shows scores for neurological deficit in animals receiving chronic nimodipine administration (subcutaneously via Alzet mini pumps) between 1 and 8 days after the onset of disease; indicated by shading in the figure. Animals were assessed using a 10‐point scale where a score of 6 signifies an inability to walk, and 8 denotes hind limb paralysis. Data presented as mean ± SEM. Stated *p* values reflect the outcome following a 2‐way repeated measures ANOVA in A and B. *p < 0.05, **p < 0.01, ***p < 0.001; Bonferroni’s multiple comparison test, A: EAE + drug n = 20, EAE + vehicle n = 17; B: EAE + drug n = 13, and EAE + vehicle n = 13). ANOVA = analysis of variance; EAE = experimental autoimmune encephalomyelitis. [Color figure can be viewed at www.annalsofneurology.org]

Given that a bolus of nimodipine reversed spinal hypoxia in EAE, and that hypoxia in EAE is known to cause neurological deficits,^5^ we next explored whether nimodipine is able to restore neurological function in EAE. Animals with EAE on the second or third day of disease expression were randomly allocated to receive a single dose of either nimodipine (n = 30; 30 mg/kg) or vehicle (n = 18) via oral gavage. We found that nimodipine improved function in the majority of animals. Moreover, the beneficial effects of a single oral dose of nimodipine extended over several days, with nimodipine‐treated animals showing significantly less hind limb dysfunction than vehicle‐treated animals (Fig 5A; 2‐way ANOVA with Bonferroni correction, *F* (4, 230) = 3.49, *p* = 0.009), particularly at day 2 (*p* < 0.01; 95% CI = 0.197–2.08), day 3 (*p* < 0.001; 95% CI = 0.797–2.68) and day 4 (*p* < 0.001; 95% CI = 0.684–2.57). Importantly, animals treated with nimodipine were able to walk 4 days following a single dose, compared with animals that received vehicle, which were unable to walk (Fig 5A): this is an especially important improvement in clinical terms. We also found that the treated rats showed a small *increase* in core body temperature (+0.73°C ± 0.17°C), perhaps due to their increased physical activity, and so the improvement in function could not be due to body cooling.

To determine whether prolonged administration of the drug would result in additional benefit, animals were implanted with subcutaneous slow release mini‐pumps containing either nimodipine (loaded to release 30 mg/kg/day for 7 days; n = 13) or vehicle (n = 13). Using the 10‐point scale, we found that most (67%) animals given nimodipine were able to walk throughout the first peak of disease, whereas the majority of (77%) animals administered vehicle exhibited a normal disease course, with hind limb paralysis at a similar stage of disease (Fig 5B). In particular, animals treated with nimodipine showed a significantly lower score for neurological deficit on days 3 (*p* < 0.05; 95% CI = 0.160–3.840) and 4 post‐onset of disease (*p* < 0.05; 95% CI = 0.160–3.840). The beneficial effects of nimodipine persisted even after the pumps were removed at 7 days, such that most (69%) drug‐treated animals retained some hind limb function, whereas most (77%) vehicle‐treated animals exhibited bilateral hindlimb paralysis, and remained paralyzed at termination. Over the entire disease course, treatment with nimodipine was able to confer a significant (*p* < 0.0001) degree of protection against loss of function. We additionally found that animals treated with nimodipine possessed significantly (*p* < 0.05) more gastrocnemius muscle mass at termination (nimodipine, 0.67 ± 0.05 g vs vehicle, 0.54 ± 0.03 g).

### *Chronic Therapy with Nimodipine Reduces Demyelination in both EAE and a Model of the Early MS Lesion*

**Figure 6:**
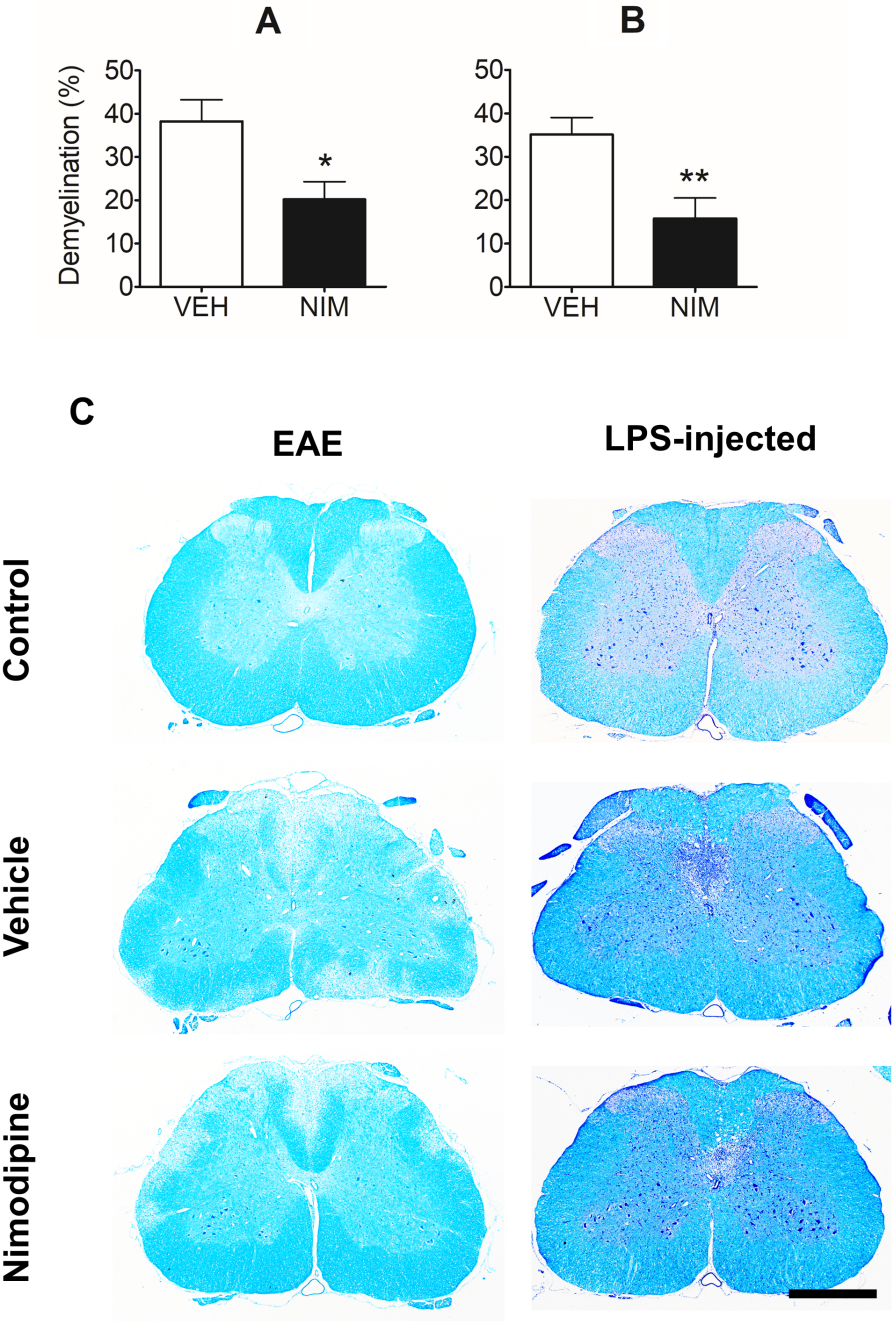
The effect of nimodipine on demyelination. Grouped data with representative micrographs are presented. (A) Shows the percent demyelination across the entire spinal cord section in animals with EAE compared with controls, whereas graph (B) shows the percentage demyelination of the dorsal columns in LPS‐injected animals, following treatment with either vehicle or nimodipine. Representative sections from control (top) and EAE animals (left), or LPS‐injected animals (right), treated with vehicle (middle) or nimodipine (bottom) labeled with LFB (left) or LFB/PAS/H (right) are shown in (C) where the paucity of blue coloration in the white matter denotes demyelination. *p < 0.05, **p < 0.01; Mann–Whitney *U* test. Scale bars 0.5 mm. EAE = experimental autoimmune encephalomyelitis; LFB/PAS/H = Luxol fast blue, periodic acid Schiff, and hematoxylin; LPS = lipopolysaccharide; NIM = nimodipine; VEH = vehicle.

Histological examination of the lumbar spinal cord of animals with EAE at 28 days postimmunization revealed that animals chronically (7‐day mini pump) treated with nimodipine exhibited significantly (Mann–Whitney *U* test, *U* = 39.0, *Z* = −2.33; *p* < 0.021) less demyelination than vehicle‐treated controls (vehicle n = 13: 38.3 ± 4.97% cross‐sectional area of the spinal white matter, nimodipine n = 13: 20.2 ± 4.06% cross‐sectional area of the spinal white matter; Fig 6A).

To determine whether nimodipine could reduce the demyelination in a spinal model of the early MS pattern III lesion,^12^ rats were administered nimodipine (30 mg/kg; i.p. immediately following surgery and p.o. subsequently; n = 20) or vehicle (n = 19) for 2 days. Histological examination of the lesion epicenter 2 weeks after lesion induction revealed that nimodipine therapy significantly reduced the magnitude of demyelination compared with controls (vehicle: 35.1% ± 3.93% cross‐sectional area of dorsal column, nimodipine: 15.8 ± 4.71% cross‐sectional area of dorsal column; Fig 6B; Mann–Whitney *U* test, *U* = 77.0, *Z* = −3.20, *p* = <0.0016). Notably, lesions failed to form in almost half of the animals treated with nimodipine (n = 9) yet were present in all controls treated with vehicle.

## Discussion

The data collectively show that loss of neurological function at the first peak of EAE is largely due to spinal hypoperfusion, hypoxia, and loss of mitochondrial function, which can result in a spinal energy crisis preventing normal function and resulting in the expression of the observed neurological deficit. Therapy with nimodipine, a CNS‐selective vasodilator,^14^ not only improves neurological function, but also protect against demyelination in EAE, and against the pattern III type of demyelination observed in early MS lesions, which we have previously demonstrated to be due to focal tissue hypoxia.^12^

To the best of our knowledge, this is the first study examining perfusion within the spinal cord in rats with EAE, and it has revealed that the expression of neurological deficit is closely related to hypoperfusion of the spinal cord, and that remission from functional deficits is accompanied by improved perfusion. Hypoperfusion is well established in MS^15^, ^16^ with delayed arterial bolus arrival time,^17^ increased mean vascular transit time,^18^ and long circulation time.^19^ It has been uncertain whether the reduced perfusion is a physiological response to reduced demand, or whether the reduced perfusion may be *causing* functional and structural damage, but the evidence that the perfusion deficits are associated with reduced cortical oxygenation^20^ indicates that the mismatch between perfusion (oxygen delivery) and oxygen demand is, therefore, responsible for causing damage. The perfusion deficits may expose vulnerable niches in the brain to such severe hypoxia that the local oligodendrocytes die, resulting in pattern III demyelination.^12^, ^15^ The evidence for hypoxia in MS is in agreement with earlier results from microarray^21^ and immunohistochemical studies.^11^ It seems reasonable that although hypoperfusion may represent the main cause of the hypoxia in early EAE, when demyelination is absent, a significant increase in oxygen demand by resident glial cells and infiltrating inflammatory cells may also contribute to the tissue hypoxia, perhaps exacerbated by demyelination at later stages of disease.

A potential cause of spinal hypoperfusion is systemic hypoperfusion, and, in MS, systemic cardiovascular dysfunction can occur as a result of autonomic disturbances, physical incapacity, inflammation, and oxidative stress.^22^ We investigated whether systemic cardiorespiratory abnormalities could account for the observed regional hypoxia but found that animals with EAE showed a broadly similar cardiovascular profile to their IFA‐treated counterparts. Thus, the severe neurological deficits, coupled with spinal hypoxia, seem to result from pathological mechanism(s) related to the spinal cord itself.

The current study supports previous findings that the inflamed spinal cord can be severely hypoxic.^5^ Furthermore, we show that the hypoxia is associated with mitochondrial failure as indicated by the ability of brief hyperoxia to increase significantly the oxidation status of mitochondrial cytochrome c oxidase in the spinal cord of animals with EAE, but not in controls. Cytochrome C oxidase is the terminal enzyme in the respiratory electron transport chain that is responsible for reduction of oxygen to water, and its activity can be limited when oxygen concentrations are low. Previous studies have shown that under low oxygen concentrations, the activity of cytochrome C oxidase becomes oxygen dependent^23^ well above the commonly reported Km. Under inflammatory conditions, such as in EAE, the consequences of hypoxia are further exacerbated by copious amounts of nitric oxide,^5^ and even nanomolar concentrations of this free radical can increase the Km of cytochrome C for oxygen and promote cell death. The current study shows that mitochondrial failure is reversible, and that function can be restored by increasing the concentration of inspired oxygen.

We have found that the inflamed spinal cord is hypoperfused, and it is reasonable to believe, first, that this is an important cause of the hypoxia and impaired mitochondrial metabolism that accompany it, and, second, that these phenomena contribute in turn to the loss of neurological function, and to demyelination (see below). Belief in this sequence of events is substantiated by our observations that nimodipine, a CNS‐specific vasodilator, not only promptly improves flow, but also tissue oxygenation, neurological function, and demyelination. The early recovery in function indicates recovery from a metabolic compromise, but the chronic improvement extending over days with sustained administration of nimodipine may involve additional mechanisms. For example, nimodipine has neuroprotective properties and can, for example, protect cultured neurons from glutamate‐induced damage,^24^ and from oxygen and glucose, and trophic, deprivation.^25^ Furthermore, nimodipine protects dopaminergic neurons from axotomy‐induced death^26^ and from damage due to 1‐methyl‐4‐phenyl‐1,2,3,6‐tetrahydropyridine (MPTP) poisoning.^27^ Nimodipine also has anti‐inflammatory properties, including significant inhibition of nitric oxide production, and of the cytokines tumor necrosis factor‐α and interleukin‐1β.^28^, ^29^ The beneficial effects of nimodipine have encouraged 2 recent studies of the effects of nimodipine in murine EAE,^30^, ^31^ and treatment was found to result in a milder disease course with reduced inflammation and demyelination, together with repair by remyelination. The possibility of spinal hypoperfusion and its downstream consequences were not considered in the earlier studies, and so the beneficial effects of nimodipine therapy on tissue hypoxia and neurological function were not considered.

The observed protection from pattern III demyelination by nimodipine is consistent with our earlier observation that pattern III demyelination is due to hypoxia and is reduced by breathing oxygen.^12^ However, the protection from demyelination observed in EAE is more surprising and it points to anti‐inflammatory actions of nimodipine (see above), perhaps in protecting or repairing the integrity of the blood‐brain barrier. The protection may also indicate that some of the demyelination in EAE is not directly mediated by autoimmunity, as often believed, but may rather be due to the consequences of hypoperfusion, including hypoxia and mitochondrial failure.

The current findings encourage consideration that nimodipine may be of value in the therapy of MS, given that patients with MS exhibit hypoperfusion^16^ and CNS hypoxia^20^ together with demyelination. Clinical use of nimodipine is facilitated by the information that the drug is already in human use to avoid complications following subarachnoid hemorrhage,^32^ and the drug has already been administered to 4 patients with MS to control paroxysmal symptoms.^33^

If used therapeutically in MS, we consider that nimodipine may have particular benefit if administered promptly at the onset of new relapses. The advantages of hyperacute therapy have been stressed in optic neuritis, a disease related to MS.^34^ We suggest that the start of the neurological deficit signals the onset of focal hypoperfusion and tissue hypoxia sufficient to cause neuro/axonal inexcitability resulting in the loss of function. Although the neurons/axons are rendered inexcitable, they remain viable so that conduction can be restored by restoration of the vascular supply. Thus, prompt administration of drugs like nimodipine can be expected to reduce the severity, and curtail the duration, of a relapse. Beyond restoring function, the current results show that therapy to improve blood flow can also be expected to protect from demyelination, thereby limiting the size of new lesions.

Nimodipine is also indicated as a therapy for MS because the reduced cerebral perfusion in the disease occurs in the presence of raised circulating concentrations of the potent vasoconstrictive agent endothelin‐1.^35^ Nimodipine can counteract the effects of endothelin‐1, in a similar way to bosentan, which has been demonstrated to restore cerebral hypoperfusion in MS to within the control flow range.^35^

Aside from correcting hypoperfusion, it is possible to improve oxygen delivery to the CNS by increasing the hematocrit, and it is interesting in this context that therapy with erythropoietin (EPO; which has a range of effects but most notably boosts red cell production) improved outcome in both a pilot study in progressive MS and a phase II trial in optic neuritis.^36^, ^37^

We conclude that strategies to improve perfusion and oxygen delivery to the inflamed CNS may offer substantial clinical benefit in MS, and in other neurological diseases where perfusion is reduced, including Alzheimer’s disease and dementia.

## Author Contributions

K.S., R.D., and A.Da. contributed to the conception and design of the study. All authors contributed to data acquisition and analysis of the data. K.S., R.D., and A.Dy. contributed to drafting the manuscript and figures.

## Potential Conflicts of Interests

The authors declared no conflict of interest.

## Supporting information


**Movie S1.** Intravenous spinal microspheres

Movie showing fluorescent microspheres travelling in the dorsal surface veins beneath the L2 vertebra of an anaesthetized rat. Some slowly moving beads are particularly clear and are probably rolling along the surface of endothelial cells, but other beads are travelling suspended in the plasma and appear as long streaks that travel distances between frames. The velocity of the suspended beads provides a measure of the velocity of blood flow.
